# Transcriptome analysis of *Aedes aegypti* Aag2 cells in response to dengue virus-2 infection

**DOI:** 10.1186/s13071-020-04294-w

**Published:** 2020-08-17

**Authors:** Man-jin Li, Ce-jie Lan, He-ting Gao, Dan Xing, Zhen-yu Gu, Duo Su, Tong-yan Zhao, Hui-ying Yang, Chun-xiao Li

**Affiliations:** State Key Laboratory of Pathogen and Biosecurity, Beijing Institute of Microbiology and Epidemiology, Beijing, 100071 China

**Keywords:** *Ae. aegypti*, Aag2, DENV2, RNAseq, Transcriptome

## Abstract

**Background:**

Dengue virus (DENV) is a flavivirus transmitted by mosquitoes that is prevalent in tropical and subtropical countries and has four serotypes (DENV1-4). *Aedes aegypti*, as the main transmission vector of DENV, exhibits strong infectivity and transmission. With the aim of obtaining a better understanding of the *Ae. aegypti*-DENV interaction, the transcriptome changes in DENV-2-infected Aag2 cells were studied to describe the immune responses of mosquitoes using the *Ae. aegypti* Aag2 cell line as a model.

**Methods:**

RNAseq technology was used to sequence the transcripts of the *Ae. aegypti* Aag2 cell line before and after infection with DENV-2. A bioinformatics analysis was then performed to assess the biological functions of the differentially expressed genes, and the sequencing data were verified by quantitative reverse transcription-polymerase chain reaction (qRT-PCR).

**Results:**

The transcriptome analysis generated 8866 unigenes that were found in both groups, 225 unigenes that were only found in the infection group, and 683 unigenes that only existed in the control group. A total of 1199 differentially expressed genes, including 1014 upregulated and 185 downregulated genes, were identified. The bioinformatics analysis showed that the differentially expressed genes were mainly involved in the longevity regulating pathway, circadian rhythm, DNA replication, and peroxisome, purine, pyrimidine, and drug metabolism. The qRT-PCR verification results showed the same trend, which confirmed that the expression of the differentially expressed genes had changed, and that the transcriptome sequencing data were reliable.

**Conclusions:**

This study investigated the changes in the transcriptome levels in the DENV-2-infected *Ae. aegypti* Aag2 cell line, which provides a faster and effective method for discovering genes related to *Ae. aegypti* pathogen susceptibility. The findings provide basic data and directions for further research on the complex mechanism underlying host-pathogen interactions.
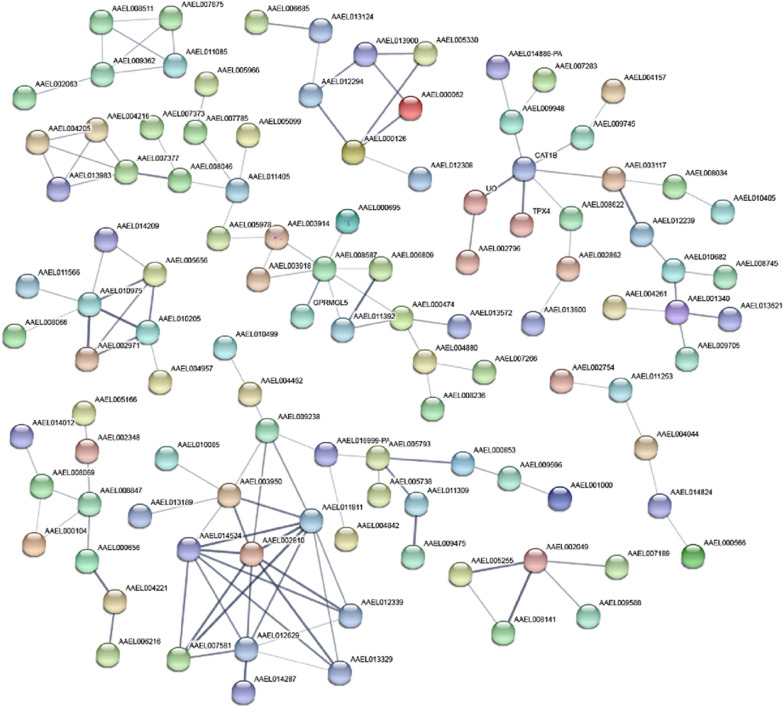

## Background

Dengue virus (DENV) is a single-stranded positive-strand RNA virus that can be divided into four serotypes (DENV1-4), and among these serotypes, DENV-2 is the most widely transmitted and infects millions of people each year [1]. DENV is transmitted mainly through *Aedes* mosquitoes, and *Aedes aegypti* (Linnaeus) is its main transmission vector. In mosquitoes, infection with arboviruses will generate an innate immune response and thereby induce changes in the gene expression profile and complex host-pathogen interaction mechanisms. Arbovirus infections usually cause acute pathological symptoms in the human body, which are usually relatively mild in the vector [2, 3]. The related regulation of the virus infection is complex in mosquitoes and differs between different tissues and parts of the body. Since Grace [4] first established an insect cell line from moths, many insect cell lines have been used as important tools for studying insects. Because cell lines are homologous, sensitive and repeatable, subtle changes in response to different types of pathogens and stimuli can be detected. The *Ae. aegypti* Aag2 cell line is an embryonic-derived cell line [5–7] that exhibits immune activity and responses very similar to those found in *Ae. aegypti* individuals [8, 9]. In addition, virus can continuously replicate in the Aag2 cell line, which indicates that this cell line can be used for various *in vitro* pathogen-related experiments [9–12]. Therefore, the Aag2 cell line can be used as a rapid and convenient tool for studying the immune responses of *Ae. aegypti* [13].

Since the sequencing of the *Ae. aegypti* genome was completed in 2007 [14], researchers have identified putative orthologous genes based on the immune genes of *Drosophila melanogaster* (Meigen) and *Anopheles gambiae* (*sensu stricto*) and have gained a more comprehensive understanding of the *Ae. aegypti* genes. The transcriptome, which comprises the set of gene transcripts expressed by cells, tissues or organisms under specific conditions, links the genetic information encoded in the genome with the biological function of the proteome [15, 16]. The development of high-throughput sequencing technology has paved the way for the development of approaches for effectively understanding and analyzing the interactions between host and virus and helps provide more transcription-level information for the design of novel strategies for blocking virus transmission.

In this study, to identify genes related to dengue virus infection, we used RNAseq technology to analyze the changes in the transcriptome of the *Ae. aegypti* Aag2 cell line induced by DENV-2 infection and validated some of the differentially expressed mRNA transcripts by quantitative reverse transcription-polymerase chain reaction (qRT-PCR). Through analyses of the interactions between DENV-2 and *Ae. aegypti*, we sought to discover key transcriptional regulatory factors in this process and block viral replication and transmission in mosquitoes.

## Methods

### Cells and virus

In this study, *Ae. aegypti* Aag2 cells were passaged in Schneider’s *Drosophila* Medium (SDM; Gibco, Grand Island, NY, USA) containing 10% fetal bovine serum (FBS; Gibco), cultured at 28 °C in an incubator with 5% CO_2_. BHK-21 cells were passaged in Dulbecco’s modified Eagle’s medium (DMEM; Gibco) containing 10% FBS, cultured at 37 °C in an incubator with 5% CO_2_, and used for determination of the virus titer. The DENV-2 Guangdong strain, which was provided by Guangdong Provincial Centers for Disease Control and Prevention, was passaged by the brains of suckling mice and used for infection. The virus titer was measured through plaque assays using monolayer BHK-21 cells.

### Virus infection

Five milliliters of Aag2 cells at a density of 1 × 10^6^ cells/ml was seeded into 25-cm^2^ cell flasks. After 24 h of incubation, DENV-2 was diluted to an appropriate ratio in SDM medium containing 2% FBS and 0.1% antibiotics to obtain a multiplicity of infection (MOI) of 1. One milliliter of the diluted virus was added to each cell flask and mix gently by shaking for 15 min, and these steps were repeated 4 times. After 1 h of infection, the virus suspension was decanted, and 5 ml of SDM medium containing 2% FBS and 0.1% antibiotics was added to each bottle. After 4 days, the cells were sampled for transcriptome sequencing, and the supernatant was tested for virus. Four biological replicates were obtained from the infection and control groups.

### Plaque assays

To verify the replication of DENV-2 in Aag2 cells, supernatant and cell samples were obtained at 1, 2 and 4 days post-infection (dpi), and 4 biological replicates were collected at each time point. The supernatant and cell samples obtained at 1 dpi were subjected to 3 freeze (− 80 °C)-thaw cycles and serially diluted (10^0^, 10^−1^, 10^−2^ and 10^−3^). The samples collected at 2 and 4 dpi were diluted 10^−1^, 10^−2^, 10^−3^ and 10^−4^ after the same freeze-thaw operations and added to a 12-well plate covered with a layer of BHK-21 cells using the same above-mentioned steps for virus infection. One milliliter of DMEM (2×, Gibco) and 2.5% low-melting agar (Sigma-Aldrich, St. Louis, MO, USA) containing 0.1% antibiotics at a 1:1 ratio was added to each well. Three replicates of each gradient were included in the analysis. After coagulation, the cells were incubated at 37 °C in an incubator with 5% CO_2_ for 7 days, and 12 ml of 1% crystal violet (Sinopharm Chemical Reagent, Shanghai, China) and 1 ml of 4% formaldehyde (Xilong Chemical Reagent, Guangdong, China) were added to each well. After staining for 2 h, the solution of crystal violet was recovered, the agar block was slowly buffered, and after drying, the number of plaques was calculated as plaque forming units per milliliter (PFUs/ml). qRT-PCR (Probe) was also used to detect the virus copy number in the supernatant and cells, and a DENV-2 nucleic acid extraction detection kit (Suneye Biotechnology, Beijing, China) was used.

### RNA extraction, cDNA library construction and sequencing

Total RNA was extracted with TRIzol (Invitrogen, Carlsbad, CA, USA) and dissolved in 50 µl of RNase-free H_2_O (Takara, Tokyo, Japan). Sequencing libraries were generated using the NEBNext^®^ ultraTM RNA Library Prep Kit for Illumina^®^ (New England Biolabs, Beverly, MA, USA). Oligo (dT) magnetic beads were used for the enrichment of mRNAs with a poly-A tail, and the first-strand cDNAs were synthesized using the M-MuLV reverse transcriptase system (New England Biolabs) with fragment mRNAs as the template and random oligonucleotides as the primers. The RNA strands were then degraded with RNase H, and second-strand cDNAs were synthesized using the DNA polymerase I system (New England Biolabs) with dNTPs as the raw material. The purified double-strand cDNA was repaired, and A-tails were added to the ends. The cDNAs were then connected with a sequencing connector and screened for the identification of 250–300-bp cDNAs using AMPure XP beads (New England Biolabs). The identified cDNAs were amplified by PCR, the PCR products were purified with AMPure XP, and a library was then obtained. After construction of the cDNA library, the library was initially quantified using a Qubit2.0 fluorometer (Thermo Fisher Scientific, Waltham, MA, USA) and diluted to 1.5 ng/µl, and the insert size of the library was then detected using an Agilent 2100 Bioanalyzer (Agilent Technologies, Santa Clara, CA, USA). If the insert size met the expectation, the effective concentration of the library (the effective concentration of the library was higher than 2 nm) was accurately quantified by qRT-PCR to ensure the quality of the library. If the quality of the libraries is satisfactory, different libraries were pooled according to the requirements of effective concentrations and target off-line data volume, and Illumina sequencing was then performed to generate 150-bp paired terminal reads. The basic principle of the sequencing analysis was sequencing by synthesis.

### Bioinformatics analyses

First, the raw data were filtered, which mainly included the removal of reads with adapters, reads containing base information that cannot be determined), and low-quality reads (the base number of Qphred ≤ 20 accounts for more than 50% of the whole read length). HISAT2 software was then used to rapidly and accurately compare the clean reads with the reference genome to obtain information on the position of the reads on the reference genome [17]. The Integrated Genomics Viewer browser was used to visually browse the information by combining the species reference genome and the annotation file. The new transcripts were then assembled using String Tie software [18], and database annotations, such as Pfam, SUPERFAMILY, GO and KEGG annotations, were performed. Using the feature counts tool in the Subread software [19], the gene expression levels in each sample were quantitatively analyzed. After the expression matrix was obtained, the sequencing depth and gene length were corrected to obtain the number of fragments per kilobase of transcript sequence per million base pairs sequenced (FPKM values) [20, 21]. According to the FPKM values, Pearson correlation coefficients for the correlations within and between groups were calculated, and a heat map was drawn. A principal components analysis (PCA) was then performed to evaluate the differences between groups and the repetition of samples within groups. The differences were then analyzed using DESeq2 [22, 23]. The criteria used for the screening of differentially expressed genes (DEGs) were | log_2_ (fold change (FC)) | > 1 and Padj < 0.05. Gene Ontology (GO) functional enrichment analysis and Kyoto Encyclopedia of Genes and Genomes (KEGG) pathway enrichment analysis were performed using ClusterProfiler software [24, 25]. The STRING protein–protein interaction (PPI) database was used to analyze the interaction network of the differentially expressed proteins [26].

### Quantitative real-time PCR validation

The 12 genes showing significant differential expression were verified by qRT-PCR (Dye). The gene IDs of these 12 genes are LOC5578354 (AAEL003508), LOC5564126 (AAEL004097), LOC5564263 (AAEL004174), LOC5572842 (AAEL010062), LOC5576631 (AAEL012655), LOC5564671, LOC5575760 (AAEL002721), LOC5578712 (AAEL013828), LOC110674007, LOC5573688 (AAEL013344), LOC5575353 (AAEL002583) and LOC5567757 (AAEL000126). The cell samples were prepared using the same above-described procedure. Four biological replicates were obtained at 1, 2, and 4 dpi from the infected group and uninfected group. The sequences of the gene primers used in the qRT-PCR analysis are shown in (Additional file 1: Table S1). The qRT-PCR reaction system included 1.0 µl of template (cDNA), 0.3 µl of 10 mM primer F/R (Tian Yi Huiyuan Bioscience & Technology, Beijing, China), 5 µl of Premix Taq (Takara), 0.5 µl of EvaGreen (Biotium, San Francisco, CA, USA), and 2.9 µl of RNase-free H_2_O (Takara). The reaction conditions were 45 cycles of 95 °C for 5 min, 95 °C for 10 s, and 60 °C for 30 s, and the melting curve was obtained from 65 °C to 95 °C. The reactions were performed using the Roche LightCycler^®^ 480II real-time PCR system (Roche, Basel, Switzerland), and 3 technical replicates of each sample were included in the analysis. Actin-5C and ribosomal protein S6 (RPS6) were used as reference genes, and the relative expression levels (FCs) in DENV-2-infected Aag2 cells were calculated using the 2^-ΔΔCT^ method and are expressed as FCs relative to the levels in uninfected cells, which were used as a control [27, 28].

## Results

### Infection of Aag2 cells with DENV-2

Aag2 cells were continuously observed for 4 days after DENV-2 infection. The virus-infected cells exhibited a nonlytic infection with no morphology and no cytopathic effect (CPE) compared with uninfected cells. Cell and supernatant samples were collected at 1, 2 and 4 dpi, and plaque assays were performed to detect the virus titers in these samples. The virus titers in the cell samples obtained at 1, 2 and 4 dpi were 0, 3.20 × 10^2^ and 4.83 × 10^2^ PFUs/ml, respectively, and those in the corresponding supernatant samples were 0.24 × 10^2^, 6.40 × 10^2^ and 62.55 × 10^2^ PFUs/ml. The virus copy numbers in the cell and supernatant samples collected at 1, 2 and 4 dpi were simultaneously detected by a qRT-PCR (Probe) analysis. The results showed that the virus copy numbers in the cell samples collected at 1, 2 and 4 dpi were 3.28, 2.94 and 3.38 (Log_10_), respectively, and those in the supernatant samples were 4.27, 4.44 and 5.14 (Log_10_), respectively. In this study, cell samples collected at 4 dpi were selected for transcriptome sequencing.

### Transcriptome alteration in Aag2 cells infected with DENV-2 and sample biological repetition

**Fig. 1:**
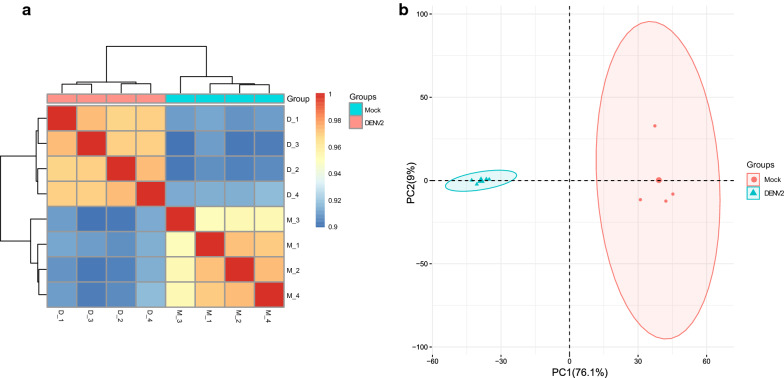
Heatmap of the correlations between samples (**a**) and the principal components analysis (**b**). Mock refers to the uninfected group, and DENV2 is the DENV-2 infected group. Four biological replicates of each group were included in the analyses

Transcriptome sequencing of cell samples obtained at 4 dpi was performed to analyze and verify the transcriptome changes induced in Aag2 cells by infection with DENV-2. Four biological replicates of each group were included in the analysis, and the results identified 51,096,080, 43,125,874, 42,338,962 and 46,677,830 raw reads in the four repeats from the infection group and 48,724,088, 51,748,932, 50,600,756 and 55,023,262 in the four repeats from the control group. After data filtering, 50,436,036, 42,663,786, 41,621,178 and 45,731,474 clear reads were obtained from the infected group, and 47,885,880, 50,965,704, 49,833,962 and 54,099,464 were found for the four repeats from the control group. Among these reads, 8866 unigenes were found in both the infection and control groups, 225 unigenes were only found in the infection group, and 683 unigenes were found only in the control group. A total of 1689 new annotated genes were predicted. The differences between the infection and control groups and the repeated samples within the groups are shown in an inter-sample correlation heat map (Fig. 1a) and PCA charts (Fig. 1b). The results showed that the differences between the samples were more obvious, and the samples within the groups exhibited better repeatability.

### Bioinformatics analysis of differentially expressed genes

**Fig. 2:**
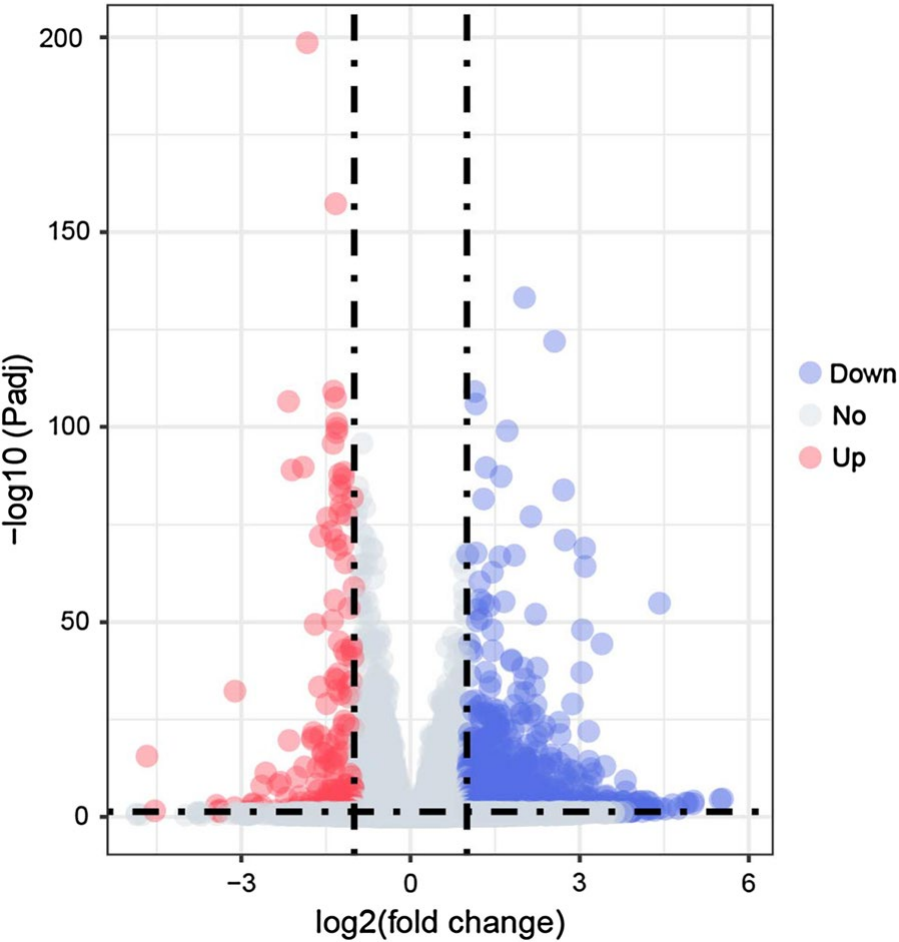
Volcano map of differentially expressed genes. The differentially expressed genes were selected based on the criteria Log_2_ (FC) > 1 and Padj < 0.05. The red dots represent the 185 upregulated genes, legend is “Up”. The blue dots represent the 1014 downregulated genes, legend is “Down”. The grey dots represent the unchanged gene, legend is “No”

A DESeq2 analysis using | log_2_ (FC) | > 1 and Padj < 0.05 as the screening criteria identified 1199 DEGs that might be related to DENV-2 infection (Additional file 2: Table S2). Among these DEGs, 1014 genes were downregulated, and the Log_2_ (FC) values were between − 5.6 and − 1. In contrast, 185 genes were upregulated, and the Log_2_ (FC) values were between 1 and 4.7 (Fig. 2). To further analyze the related functions of DEGs and identify biological pathways that play a key role in biological processes with the aim of revealing and understanding the basic molecular mechanism, we performed enrichment analyses of the 1199 DEGs.

**Fig. 3:**
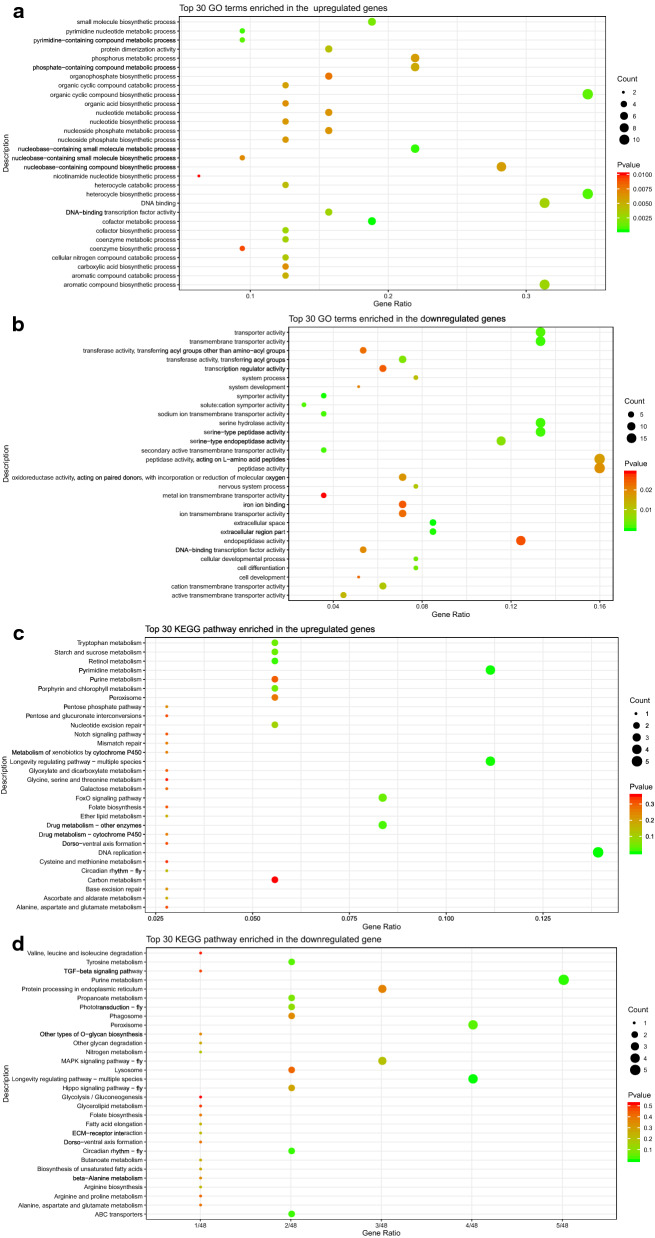
Bubble map of the top 30 most enriched GO terms and KEGG pathways. **a** Top 30 GO terms enriched in the upregulated genes. **b** Top 30 GO terms enriched in the downregulated genes. **c** Top 30 KEGG pathways enriched in the upregulated genes. **d** Top 30 KEGG pathways enriched in the downregulated genes. Y-axis label represents the distinct GO terms or KEGG pathways, and X-axis label represents the Gene Ratio. Gene Ratio refers to the ratio of DEGs annotated in the term or pathway to total number of genes annotated in the term or pathway. The size of the bubble represents the number of enriched DEGs (big size indicates large number of enriched genes). The color represents the *P*-value of the enrichment (green color indicates high enrichment)

The GO enrichment results identified 552 terms, and these included 303 biological process (BP) terms, 65 cellular component (CC) terms, and 184 molecular function (MF) terms. Among the BP terms, the DEGs were most significantly enriched in metabolic processes such as those involving pyrimidine nucleotides, pyrimidine-containing compounds, and ribonucleosides, and catabolic processes such as those involving cellular nitrogen compounds, heterocycles, and aromatic compounds. In addition, the identified DEGs are also involved in cell development and differentiation, biosynthesis, and signaling pathways, among other processes. The analysis of the CC terms revealed that the DEGs were most significantly enriched in cellular structures such as the extracellular space, extracellular region part, and plasma membrane, and participate in the formation of synapses, the transmembrane transporter complex, various organelles, and the cytoskeleton. Among the MF terms, DEGs were most significantly enriched in the processes related to DNA-binding transcription factor activity, transcriptional regulator activity, symporter activity, transmembrane transporter activity, and transferase activity, among others. In addition, the DEGs were found to participate in the regulation of the binding of various substances, such as iron ions, cofactors, and NADP, and of the activities of enzymes such as oxidoreductases, peptidases, hydrolases, and synthases. Separate enrichment analyses of the upregulated and downregulated DEGs were then performed. The upregulated genes were mainly enriched in the metabolic processes of cofactors, nucleobase-containing small molecules, and pyrimidine nucleotides, and in the biosynthesis processes of heterocyclic, organic cyclic compounds, and small molecules (Fig. 3a, Additional file 3: Table S3). In contrast, the downregulated genes were mainly enriched in the formation of the extracellular space and extracellular region part and regulate the processes related to symporter activity, transmembrane transporter activity, serine-type enzyme activity and other various enzyme activities as well as cell development and differentiation (Fig. 3b, Additional file 4: Table S4).

The KEGG enrichment results showed that the DEGs were enriched in 67 pathways and mainly participated in longevity regulating pathway-multiple species, circadian rhythm-fly, DNA replication, purine metabolism, pyrimidine metabolism, peroxisome, drug metabolism-other enzymes and other pathways. The upregulated DEGs were enriched in 39 pathways, which mainly included DNA replication, pyrimidine metabolism, longevity regulating pathway-multiple species, retinol metabolism, and FoxO signaling pathway (Fig. 3c, Additional file 5: Table S5). In contrast, the downregulated DEGs were enriched in 41 pathways, which mainly included longevity regulating pathway-multiple species, purine metabolism circadian rhythm-fly, ABC transporters, tyrosine metabolism, MAPK signaling pathway-fly and others (Fig. 3d, Additional file 6: Table S6).

**Fig. 4:**
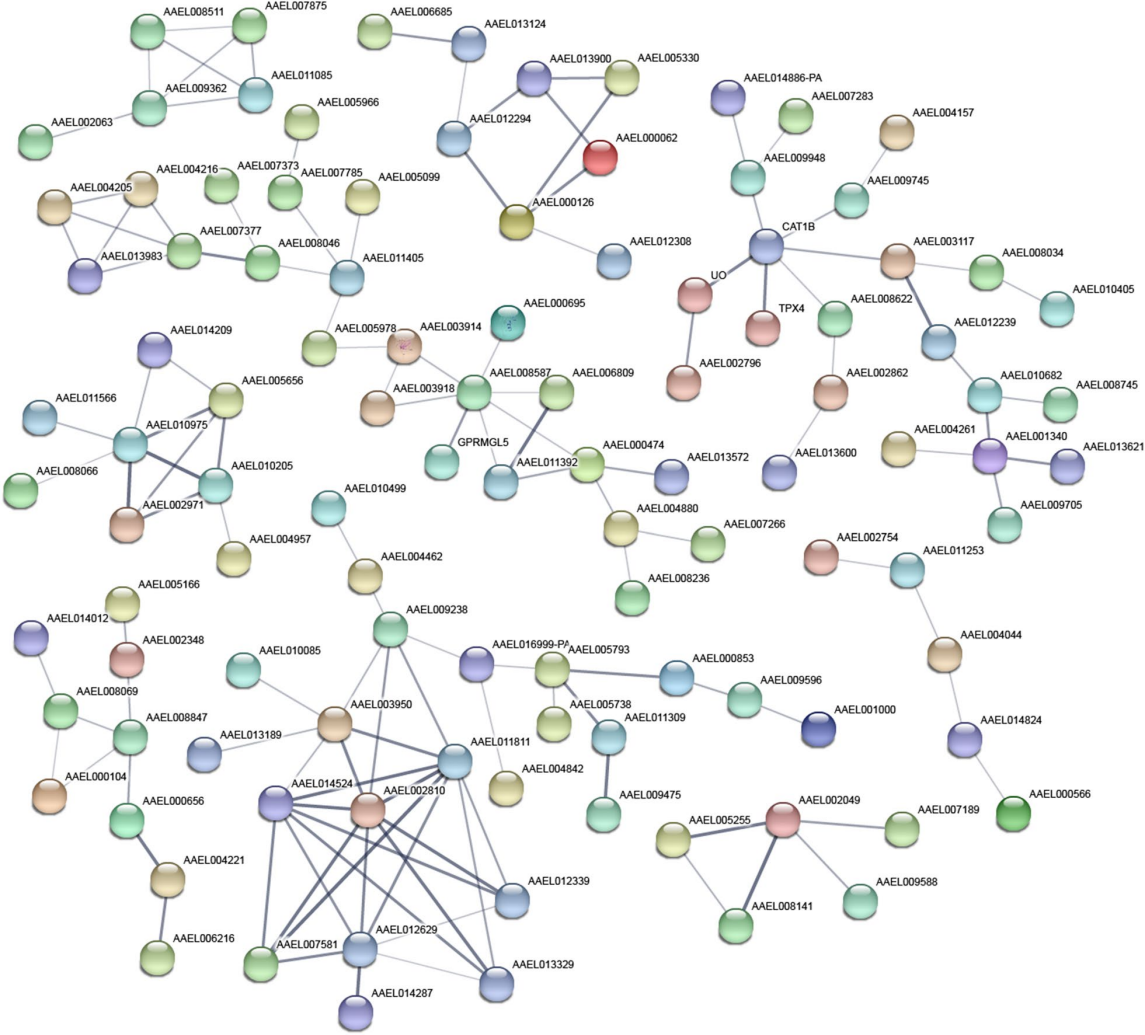
Protein–protein interaction analysis. The analysis of 550 differentially expressed protein-coding genes revealed 108 interacting proteins with more than two edges, as shown in the maps. The connecting lines represent predicted functional associations. The thickness of the lines shows the strength of the data support

Using the STRING database, PPIs analyses of 550 genes with protein-coding functions among the 1199 DEGs, and 108 genes with more than two connecting lines were identified (Fig. 4). In addition, 132 edges (representative of PPIs) were obtained, and the PPI enrichment *P*-value was less than 1.0e−16. Nine groups of interacting proteins were obtained from the DEGs with protein-coding functions.

### qRT-PCR validation of sequencing data

**Fig. 5:**
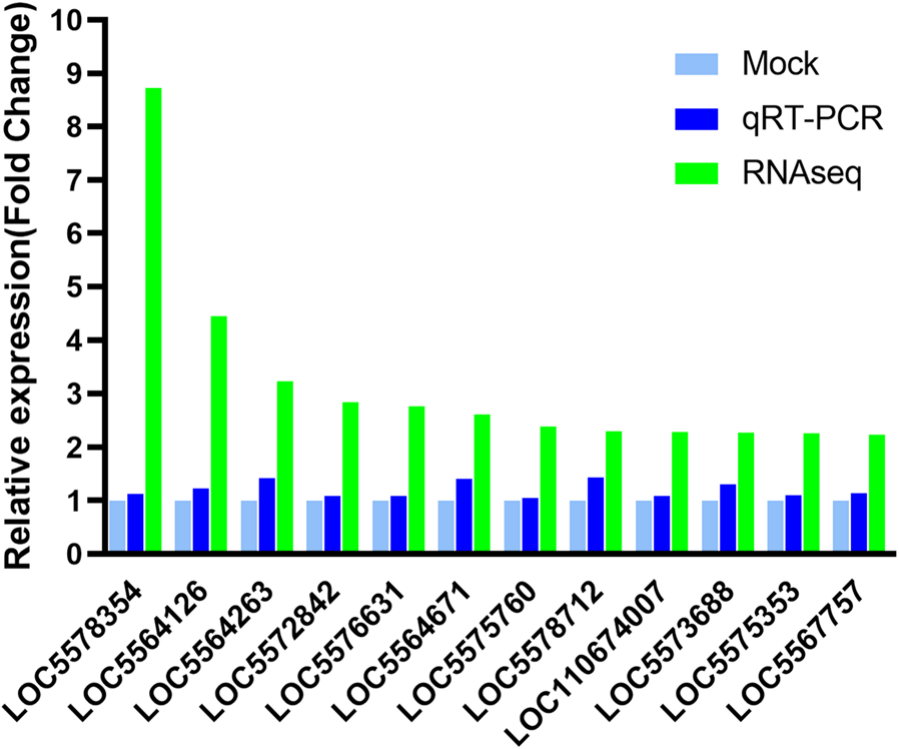
qRT-PCR verification of 12 differentially expressed genes identified by RNAseq. Mock refers to an untreated sample, and the default gene expression was unchanged, and the FC value was set to 1. The relative expression level (FC) of a gene in DENV-2-infected Aag2 cells was calculated using the 2^-ΔΔCT^ method and presented as fold changes relative to the expression in uninfected cells

To verify the transcriptome sequencing data, we selected 12 genes with significant differential expression and used cell samples collected at 4 dpi for qRT-PCR verification. The relative expression levels of the 12 genes obtained by qRT-PCR showed FC in the range of 1.05 to 1.43. Specifically, among these 12 genes, the T-box transcription factor TBX6 (TBX6, LOC5564263, AAEL004174), proteasome maturation protein (LOC5564671), and methylthioribose-1-phosphate isomerase (M1Pi, LOC5578712, AAEL013828) were significantly increased after infection (*P* < 0.05), with FCs of 1.41, 1.41 and 1.43, respectively (Fig. 5).

### Changes in gene expression at 1, 2 and 4 dpi

To observe the changes in the expression levels of the 12 above-described genes in cells, we also performed qRT-PCR experiments at 1 and 2 dpi. The FC range at 1 dpi was 0.77–1.22; among the 12 genes, Toll-like receptor 7 (TOLL7, LOC5575353, AAEL002583) exhibited a significant increase in expression after infection (*P* < 0.05), with a FC of 1.22, and proteasome maturation protein and protein extramacrochaetae (PEM, LOC5575760, AAEL002721) showed significant decreases in expression after infection (*P* < 0.05), with FC values of 0.88 and 0.84, respectively. The FC range at 2 dpi ranged from 0.74 to 1.19, and among the 12 genes, methylthioribose-1-phosphate isomerase exhibited a significant increase in expression after infection (*P* < 0.05), with a FC of 1.16. The FC values for genes at the different time points were also analyzed by one-way ANOVA, and the results are shown in Table 1. Among the investigated genes, serine pyruvate aminotransferase (LOC5578354, AAEL003508), proteasome maturation protein, methylthioribose-1-phosphate isomerase, protein lethal (2) essential for life (LOC5573688, AAEL013344) exhibited significant differentially expression at the different time points after infection (*P* < 0.05), and all of these were downregulated at the early stage of infection and upregulated at the later stage.

**Table 1:** Expression levels of 12 genes at 1, 2 and 4 dpi

Gene	1 dpi		2 dpi		4 dpi		ANOVA	
	FC	*P*	FC	*P*	FC	P	*F*	*P*
LOC5578354	0.772	0.136	1.191	0.340	1.128	0.436	5.179	0.032
LOC5564126	1.183	0.124	1.155	0.333	1.224	0.087	0.345	0.717
LOC5564263	1.090	0.184	1.163	0.191	1.415	0.009	3.703	0.067
LOC5572842	0.974	0.607	0.992	0.975	1.091	0.549	0.464	0.643
LOC5576631	1.059	0.506	1.019	0.842	1.083	0.519	0.158	0.856
LOC5564671	0.879	0.025	1.131	0.053	1.407	0.043	4.850	0.037
LOC5575760	0.840	0.038	1.129	0.283	1.050	0.790	2.082	0.181
LOC5578712	0.895	0.105	1.163	0.029	1.433	0.003	20.088	< 0.0001
LOC110674007	0.895	0.056	1.107	0.292	1.086	0.369	2.161	0.171
LOC5573688	0.899	0.209	0.740	0.237	1.297	0.143	5.284	0.030
LOC5575353	1.222	0.038	1.118	0.389	1.098	0.096	0.572	0.584
LOC5567757	1.043	0.323	1.058	0.400	1.131	0.069	0.467	0.641

**Fig. 6:**
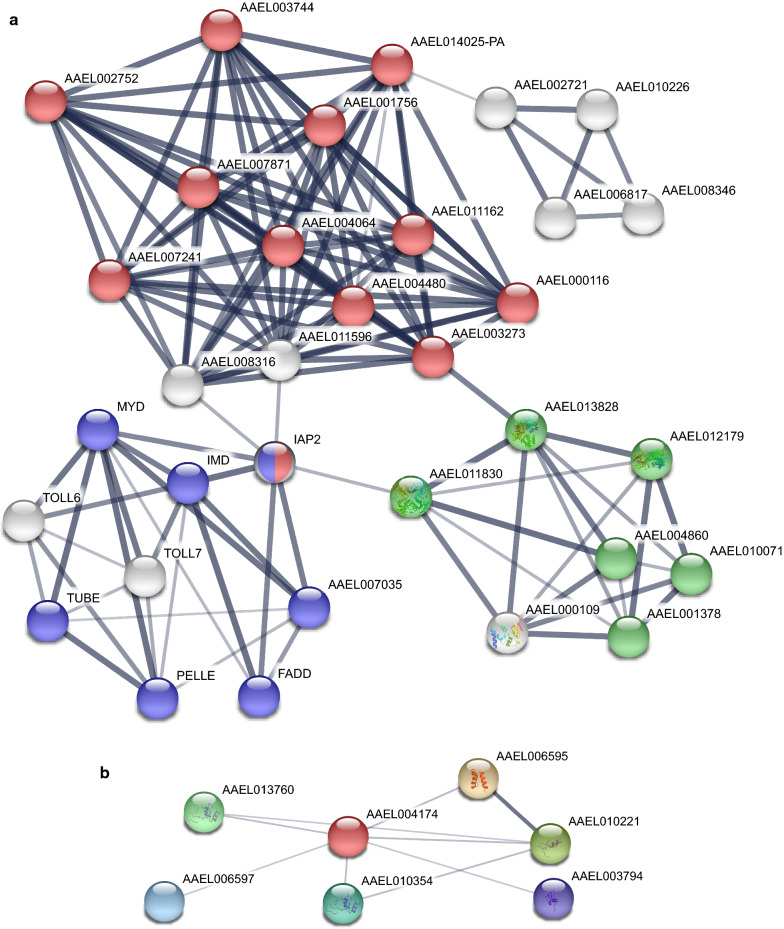
Protein–protein interaction analysis of significantly differentially expressed genes validated by qRT-PCR. **a** The PPI analyses of AAEL002721, AAEL013828 and AAEL002583 (the KEGG pathways are distinguished by different colors: red, ubiquitin-mediated proteolysis; blue, Toll and Imd signaling pathways; and green, cysteine and methionine metabolism). **b** The PPI analyses of AAEL004174. The connecting lines represent the predicted functional associations. The thickness of the lines indicates the strength of the data support

A PPI analysis of four coding genes (PEM, M1Pi, TOLL7 and TBX6) that were found to be significantly differentially expressed in the qRT-PCR analysis was then performed using the STRING database, and a total of 41 related genes were identified (Fig. 6). The GO enrichment analysis of these genes in terms showed that they are mainly enriched in several BP (L-methionine salvage from methylthioadenosine, methionine metabolic process, sulfur amino acid biosynthetic process, sulfur amino acid metabolic process, and drug metabolic process), CC (cytoplasm and nucleus) and MF (catalytic activity and metal ion binding) terms. The KEGG enrichment results indicated that the related genes are mainly involved in ubiquitin-mediated proteolysis, the Toll and Imd signaling pathways, cysteine and methionine metabolism, apoptosis-multiple species, beta-alanine metabolism, arginine and proline metabolism, glutathione metabolism, and metabolic pathways.

## Discussion

In this study, to avoid the effects of RNA degradation and accumulation on physiological functions and to ensure the quality of the cDNA library, the selected samples were collected at four dpi that exhibited high intracellular and extracellular virus titers and high virus copy numbers after DENV-2 infection for transcriptome sequencing. The analysis identified 1199 DEGs (185 upregulated and 1014 downregulated), which initially indicated that these genes might be related to DENV-2 infection. The results from the enrichment analyses demonstrated that the DEGs mainly participate in the pathways related to material metabolism, synthesis, transport, DNA replication and signaling. The PPI analysis identified the interactions within the proteins expressed in Aag2 cells during DENV-2 infection (Fig. 5). And further analysis of this results showed that the DEGs were also mainly enriched in the above pathways. The effects of DENV-2 infection on the physiological functions of Aag2 cells were further elucidated, but the specific functions need to be further explored and verified.

### Analysis of metabolic pathways

Cells change their metabolic pathways to ensure their survival after infection. In this study, the metabolic pathways that were enriched by the upregulated genes were pyrimidine metabolism, retinol metabolism, and so on. Previous studies have shown that pyrimidine metabolism participates in programmed cell death (PCD) through a dynamic balance between recovery and degradation [29]. And PCD, as an intrinsic response to viral infection, has been shown to inhibit viral replication [30, 31]. In addition, changes in host cell pyrimidine metabolism after infection also affect viral replication, and previous studies have demonstrated in fibroblasts, which are viruses that utilize different pyrimidine metabolism-related strategies to promote virus replication [32]. A metabolomics study of intraerythrocytes infected with *Plasmodium falciparum* Welch also revealed the effects of changes in the pyrimidine levels on pathogen infection [33]. Pathogens have evolved new metabolic strategies during evolution that provide optimal levels of catabolic metabolites for their own replication [34]. Studies related to retinol metabolism have revealed that retinol functions in the maintenance and promotion of immunity. Retinol deficiency affects physiological abnormalities and pathological changes caused by immune function.

Purine metabolism and tyrosine metabolism were identified as the metabolic pathways in which the downregulated genes were enriched. Purines and pyrimidines are indispensable to all life and have many important functions [35]. Purine compounds play an important role in the growth, proliferation and survival of all cells. And the results from the enrichment analysis indicate that the metabolic pathway, as the most enriched pathway, plays an important role in the interaction between Aag2 cells and DENV-2. The findings suggest that further studies can be based on the metabolic pathway, pay particular attention to relevant genes involved in the pyrimidine metabolic pathway, and further investigate the changes in physiological functions of host cell-virus infections. The relevant genes can be used as targets for inhibiting or even promoting their expression and thereby inhibiting or even blocking virus replication. And we may be able to use pyrimidine inhibitors or antimetabolites to inhibit and block DENV-2 replication in Aag2 cells.

### Analysis of signaling pathways

According to previous studies, PCD is an important part of the host defense system against pathogen invasion. Most signaling pathways identified in this study are involved in the regulation of viral infections by participating in PCD. And the upregulated DEGs were enriched in the FoxO signaling pathway, which is mainly involved in the regulation of cell life processes such as apoptosis, the cell cycle, and longevity. Previous studies have demonstrated that Japanese encephalitis virus (JEV) infection can reduce the expression of apoptosis-related FoxO signaling pathway genes and that the knockdown and overexpression of the FoxO gene promotes and inhibits apoptosis in JEV-infected Neuro-2a cells, respectively [36]. Pathways enriched in downregulated genes, such as the MAPK signaling pathway and Hippo signaling pathway-fly, also participate in this process. The Hippo signaling pathway regulates the proliferation, survival, and morphology of eukaryotic cells, and studies have shown that this pathway is involved in the process of ZIKV infection [37]. The MAPK signaling pathway also participates in the virus-host interaction process by regulating various physiological processes, such as cell growth, differentiation, apoptosis and death [38, 39].

In addition, the longevity regulating pathway-multiple species, which was found to be significantly enriched in both upregulated and downregulated genes, is also involved in the apoptotic process. In *D. melanogaster*, dietary restriction (DR) inactivates the PI3K/Akt/TOR signaling cascade by reducing signaling through the insulin/insulin-like growth factor signaling and target of rapamycin (IIS/TOR) pathway and thereby activates the FoxO signaling pathway, which is involved in apoptosis and survival processes [40]. During evolution, viruses have developed the ability to regulate a variety of host cell signaling pathways. The abovementioned signal pathways interact with each other and form a huge complex signal transmission network through activation or negative regulation. This provides us with new ideas that can interfere with the PCD process by regulating Aag2 cell-related signaling pathways and achieve the purpose of inhibiting or even blocking DENV-2 replication. And we suspect that the PCD process involved in these signaling pathways may be related to the nonlytic persistent infection phenotype without morphological and CPE changes in DENV-2-infected Aag2 cells, which needs further study.

### Analysis of immune pathways

Previous research on the mosquito immune response has provided evidence demonstrating that it is similar to the mammalian adaptive immune response, but the innate immune response also exerts a marked effect on the outcome of infection [41, 42]. The immunoregulatory mechanisms in mosquitoes include the RNAi, Toll, and JAK-STAT pathways [43–45]. The downregulated DEGs identified in this study were only enriched in the Toll and Imd signaling pathways and not in the JAK-STAT signaling pathway and the RNAi pathway, which suggests that the Toll and Imd signaling pathways might differ from the JAK-STAT signaling pathway and the RNAi pathway. This result is similar to the results obtained after the infection of *Ae. albopictus* Skuse cells with bluetongue virus [46, 47]. Moreover, the Toll and Imd signaling pathways were inhibited in DENV-2-infected Aag2 cells, and these findings were consistent with those from previous studies that showed that viruses such as DENV and CHIKV inhibit the Toll signaling pathway and thereby inhibit antiviral activity, which might reflect a “DENV downregulation trend” [48–50].

### Analysis of other pathways

The downregulated DEGs were significantly enriched in the circadian rhythm pathway-fly. The circadian rhythm is important not only for behavioral research but also for basic physiological processes such as immunity. Although the molecular mechanisms of these interactions are not fully understood, the effects of disrupting circadian rhythms on infections and diseases have been well documented [51]. Studies in this field have mainly been performed using the model organism *D. melanogaster*. Studies conducted by Schneider et al. [52] have shown that bacterial infections disrupt the circadian rhythm, revealing a functional relationship between circadian rhythm and innate immunity. Lee et al. [53] have shown that medical intervention strategies incorporating chronobiological considerations can enhance the innate immune response and improve the effect of combating pathogenic infections in *D. melanogaster*. These behavioral studies and the results obtained in this study provide new strategies and ideas for the immune regulation of vectors.

In addition, the significant enrichment of downregulated DEGs in peroxisome preliminarily indicates that this pathway is also involved in the interaction between Aag2 cells and DENV-2. Previous studies have shown that peroxisome-mediated metabolism is necessary for the immune response to infection in *D. melanogaster* [54]. Peroxisomes fight bacterial infections through classic innate immune signals, and studies have shown that the reduction of peroxisome function impairs the turnover of the oxidative burst necessary for fighting infection. Further research on peroxisomes and their role in the immune system is needed [55]. The downregulated DEGs were also enriched in ABC transporters. As the largest family of active transporters, previous research on ABC transporters has mostly focused on pesticide sensitivity and metabolism [56, 57], but the results of this study indicate that these transporters might also participate in the cellular immune process. All three pathways were inhibited in the DENV-2-infected Aag2 cells, which suggests that the genes related to these pathways might be involved in the host-virus interaction process and that enhancing the expression of these genes might inhibit or even block virus transmission.

### qRT-PCR verification of gene expression levels

For verification of the sequencing data, 12 genes that were found to show significantly upregulated expression were selected for qRT-PCR verification, and cell samples collected at four dpi were used in this analysis. Here, we mainly focused on genes related to immune activation and enhanced infection, and we thus selected some of the upregulated genes for validation. The results from the qRT-PCR verification analysis were consistent with the transcriptome sequencing results, and the expression levels increased after infection, which indicated that the expression of the selected genes in Aag2 cells changed after infection. In addition, to observe the changes in the expression levels of these 12 genes in cells, a qRT-PCR analysis was performed at one, two and four dpi. The results showed that genes exhibit dynamic changes during the interaction between Aag2 and DENV-2, and the expression of most of these genes was inhibited in the early stage and enhanced at the later stage (Table 1). This finding confirms that the genes might participate in the regulation of Aag2 processes in response to the virus and thus provides potential targets for blocking intracellular virus replication. Such as Toll-like receptor 7 (TOLL7), T-box transcription factor TBX6 and other genes.

Studies using *D. melanogaster* have revealed that TOLL7 are involved in the development of the dorsoventral axis, synaptogenesis, and axonal initiation and have demonstrated that these processes participate in the immune response [58, 59]. Mutations in the Toll signaling pathway significantly reduce the survival of *D. melanogaster* after fungal infection and are activated either by a distinct Imd signaling pathway or by combined activation of both Imd and dorsoventral pathways [60]. In humans, TOLL7 plays a very important role in the signal transduction pathways involved in innate immunity [61]. Further clarification of the relationship between TOLL7 activity and viral infection in mosquitoes will aid the formulation of new mosquito-borne virus intervention strategies. The TBX6 was upregulated at one, two and four dpi and significantly upregulated at four dpi (*P* < 0.05). The T-box gene is essential for the development of arthropod limbs. Eight genes in the *Drosophila* genome encode the T-box, and six of these genes are expressed in limb ontogenesis; in addition, three TBX6-related Dorsocross genes are required for epithelial remodeling during wing development [62], but their role in virus regulation needs to be further verified.

We then performed a PPI analysis of four DEGs (PEM, M1Pi, TOLL7 and TBX6), as shown in Fig. 6. PEM, M1Pi and TOLL7 are linked by a series of genes, and PEM and M1Pi are linked by cell division cycle 20 (CDC20, AAEL014025) and cell division cycle protein 23 homolog (CDC23, AAEL003273), which are two genes that control mitosis and the cell cycle. TOLL7 interacts with other genes through baculoviral IAP repeat-containing protein 7 (IAP2, AAEL006633), which is involved in regulating the inhibition of apoptosis, and then through mitotic spindle assembly checkpoint protein MAD2A (AAEL008316) and mitotic checkpoint serine/threonine protein kinases bub1 and bubr1 (AAEL011596), which are involved in cell mitosis. The genes interacting with TOLL7, including MYD, IMD, PELLE, TUBE, FADD and TOLL6, participate in the Toll and Imd signaling pathways. According to the results, these genes and their interacting genes are mainly involved in processes such as metabolism, biosynthesis, and cell development, and this finding is consistent with the results from the above-described analyses of the DEGs. TBX6 mainly interacted with the homeobox gene, which was first discovered to be the main gene in *D. melanogaster* that controls development and plays a key role in organogenesis and the regulation of cell proliferation and differentiation [63]. These results further confirm that the Toll and Imd signaling pathways play an important role in the regulation of DENV-2-infected Aag2 cells and that genes and pathways involved in regulating the cell cycle, proliferation differentiation, and apoptosis also act synergistically in this process.

## Conclusions

This study first involved an investigation of the changes in the transcriptome of *Ae. aegypti* Aag2 cells after infection with DENV-2. A total of 1199 DEGs, including 185 upregulated and 1014 downregulated DEGs, were detected, and these DEGs might be involved in antiviral responses and viral infection mechanisms in mosquito and mosquito cell lines. The bioinformatics analysis showed that DEGs are mainly involved in the pathways related to substance metabolism, synthesis, transport, and signaling, including pyrimidine metabolism, FoxO signaling pathway, longevity regulating pathway-multiple species, DNA replication, circadian rhythm-fly, peroxisome, and the Toll and Imd signaling pathways. The analysis of the DEGs induced by DENV-2 infection identified a total of nine groups of interacting proteins. Previous studies have indicated that the pathways activated in Aag2 cells after infection with DENV-2 and the functions of the related proteins are largely unknown. The results of this study will aid the identification of more transcription regulators that are susceptible to *Ae. aegypti*-associated pathogens and further studies of the complex mechanism underlying the interaction between mosquito vectors and arboviruses. Understanding the interaction between mosquito vectors and viruses, including the antiviral responses and immune evasion strategies after pathogen recognition, is important for the development of new methods and strategies for blocking virus replication and transmission in mosquito vectors.

## Supplementary information

**Additional file 1: Table S1.** Primers used for qRT-PCR validation.

**Additional file 2: Table S2.** Differentially expressed genes of Aag2 cell line from DENV-2-infected cells compared to uninfected cell.

**Additional file 3: Table S3.** GO annotations enriched in the upregulated differentially expressed genes.

**Additional file 4: Table S4.** GO annotations enriched in the downregulated differentially expressed genes.

**Additional file 5: Table S5.** KEGG Pathway enriched in the upregulated differentially expressed genes.

**Additional file 6: Table S6.** KEGG Pathway enriched in the downregulated differentially expressed genes.

## Data Availability

The datasets supporting the conclusions of this article are included within the article and its additional files. The raw data have been deposited in the Sequence Read Archive (SRA) database (https://submit.ncbi.nlm.nih.gov/subs/sra/) with accession numbers SRR12200475, SRR12200474, SRR12200473, SRR12200472, SRR12200471, SRR12200470, SRR12200469, SRR12200468.

## Abbreviations

DENV: dengue virus
RNAseq: RNA sequencing
qRT-PCR: quantitative reverse transcription-polymerase chain reaction
MOI: multiplicity of infection
dpi: day post-infection
PFU: plaque forming unit
DEG: differentially expressed gene
GO: Gene Ontology
KEGG: Kyoto Encyclopedia of Genes and Genomes
PPI: protein–protein interaction
PCD: programmed cell death
